# Differences in Nicotine Metabolism of Two *Nicotiana attenuata* Herbivores Render Them Differentially Susceptible to a Common Native Predator

**DOI:** 10.1371/journal.pone.0095982

**Published:** 2014-04-22

**Authors:** Pavan Kumar, Preeti Rathi, Matthias Schöttner, Ian T. Baldwin, Sagar Pandit

**Affiliations:** Department of Molecular Ecology, Max-Planck-Institute for Chemical Ecology, Jena, Germany; Rutgers University, United States of America

## Abstract

**Background:**

*Nicotiana attenuata* is attacked by larvae of both specialist (*Manduca sexta*) and generalist (*Spodoptera exigua*) lepidopteran herbivores in its native habitat. Nicotine is one of *N. attenuata*'s important defenses. *M*. *sexta* is highly nicotine tolerant; whether cytochrome P450 (CYP)-mediated oxidative detoxification and/or rapid excretion is responsible for its exceptional tolerance remains unknown despite five decades of study. Recently, we demonstrated that *M. sexta* uses its nicotine-induced CYP6B46 to efflux midgut-nicotine into the hemolymph, facilitating nicotine exhalation that deters predatory wolf spiders (*Camptocosa parallela*). *S. exigua*'s nicotine metabolism is uninvestigated.

**Methodology/Principal Findings:**

We compared the ability of these two herbivores to metabolize, tolerate and co-opt ingested nicotine for defense against the wolf spider. In addition, we analyzed the spider's excretion to gain insights into its nicotine metabolism. Contrary to previous reports, we found that *M. sexta* larvae neither accumulate the common nicotine oxides (cotinine, cotinine N-oxide and nicotine N-oxide) nor excrete them faster than nicotine. In *M. sexta* larvae, ingestion of nicotine as well as its oxides increases the accumulation of CYP6B46 transcripts. In contrast, *S. exigua* accumulates nicotine oxides and exhales less (66%) nicotine than does *M. sexta*. Spiders prefer nicotine-fed *S. exigua* over *M. sexta*, a preference reversed by topical or headspace nicotine supplementation, but not ingested or topically-coated nicotine oxides, suggesting that externalized nicotine but not the nicotine detoxification products deter spider predation. The spiders also do not accumulate nicotine oxides.

**Conclusions:**

Nicotine oxidation reduces *S. exigua*'s headspace-nicotine and renders it more susceptible to predation by spiders than *M. sexta*, which exhales unmetabolized nicotine. These results are consistent with the hypothesis that generalist herbivores incur costs of detoxification, which include the ecological costs of greater predation risks, in addition to the previously demonstrated energetic, physiological and metabolic costs.

## Introduction

Plant defense chemicals hamper insect herbivores' growth and reproduction. However, the ingestion of allelochemicals can enhance herbivore survivorship in nature, when they are co-opted for defense against natural enemies [1], [2]. Such defensive co-option requires a balance between detoxification and tolerance mechanisms. Herbivores can repurpose ingested xenobiotics for defense either with or without specific modifications [3], [4], [5]. Specialists by means of their evolved resistance and tolerance mechanisms are thought to co-opt host defenses, while generalists are thought to detoxify them, frequently at great energetic, metabolic and physiological costs [6], [7], [8], [9]. However, examples of xenobiotic-detoxifying specialists [10], [11] and xenobiotic-sequestering generalists [12], [13], [14] are also known [15]. Different herbivores' co-option mechanisms against a common xenobiotic have been studied and compared [16], [17], [18]; however, the empirical evidence on the ecological consequences of co-option, especially to other herbivores of the same host remains sparse.

*Manduca sexta* (Sphingidae) and *Spodoptera exigua* (Noctuidae) can severely defoliate *Nicotiana attenuata* (Solanaceae) in its native habitat (the Great Basin Desert, Utah, USA) [19]. *N. attenuata* produces the insecticidal alkaloid, nicotine, that retards the growth of these herbivores [20]. *M. sexta* is the most nicotine-tolerant organism known [21] and it has been used as a model to study nicotine toxicity and tolerance in insects. However as Appel and Martin [22] noted, studies on its nicotine metabolism have yielded contradictory results. Two major theories have been proposed. According to the first, nicotine is detoxified by oxidation; however, there is little agreement on the identity of the nicotine oxidation products. Snyder *et al*. [23], [24] reported cotinine and cotinine N-oxide (CNO), Wink & Theile [21] found nicotine N-oxide (NNO) and Morris [25] reported unidentified conjugates of 5′-hydroxynicotine as the major detoxification products. Cytochrome P450s (CYPs) were thought to be responsible for such oxidative detoxification, since they were found to be upregulated in response to the ingestion of nicotine [23], [26], [27]; however, none of the CYPs identified so far have been experimentally shown to oxidize nicotine in *M. sexta* larvae [28]. The second theory states that nicotine is rapidly excreted via the Malpighian tubules before it is modified [29], [30], [31]; consistent with this theory, we recently found that *M. sexta* larvae do not oxidize nicotine and excrete a substantial portion of ingested nicotine unmetabolized [1]. *S. exigua*'s nicotine metabolism has not been studied but the larvae of this species are known to be more susceptible. Feeding on nicotine-producing host plants results in more severe growth retardation and mortality for *S. exigua* than it does for *M. sexta* larvae [19]. Notably, the response of *N. attenuata* to these two herbivores is also different; the oral secretion of *M. sexta* larvae elicits a burst of ethylene in attacked leaves that suppresses the expression of a key nicotine biosynthetic gene *putrescine N-methyltransferase (PMT)*, whereas herbivory by *S. exigua* does not [32], [33]. Together, these reports indicate that *N. attenuata* discriminates between attack from *M. sexta* and *S. exigua* to tailor its induced nicotine defense response accordingly. This observation motivated the hypotheses that these herbivores may also respond differently to nicotine ingestion and that these differences would influence the larvae's interactions with their natural enemies.

Here, we compare the ability of these two herbivores to metabolize, tolerate and co-opt ingested nicotine for defense against a common and major nocturnal predator. We first studied the unexplored aspects of *M. sexta*'s nicotine metabolism. Snyder *et al*. [23], [24] proposed that CNO was the final product of the nicotine oxidation pathway, which could be formed either through a cotinine or an NNO intermediate with each oxidation step mediated by CYP4M1 or CYP4M3. Since we could not detect any nicotine oxide in our previous work, a study in which we discovered a novel function of the midgut expressed and nicotine-induced CYP6B46, we hypothesized that in the *M. sexta* strain of our laboratory culture, one or more oxidation steps could be inactive or absent. We examined the frass of field-collected *Manduca spp*. larvae feeding on native plants and administered each nicotine oxide separately to *M. sexta* larvae and analyzed whether the additional oxidized products of the pathway could be recovered from hemolymph and frass. We also examined the effects of these oxides on larval mass and mortality to evaluate if the oxides are less toxic to *M. sexta* than nicotine, a central prediction of the detoxification hypothesis. Next, we asked whether the ingestion of nicotine oxides up-regulates transcript levels of *Ms*CYP4M1, *Ms*CYP4M3 or *Ms*CYP6B46 in larval midguts and if such transcript up-regulation can be correlated with the *de novo* occurring oxidized products, if any. A second prediction of the oxidative detoxification theory is that *M. sexta* larvae excrete nicotine oxides more rapidly than they do nicotine; this was tested by injecting these compounds into the larval hemolymph and monitoring their discharge kinetics from the hemolymph. We determined whether *S. exigua* metabolizes nicotine differently than does *M. sexta* and with which fitness costs. *M. sexta* larvae exhale ingested nicotine to deter the nicotine-sensitive predatory wolf spider *Camptocosa parallela* (Lycosidae). This spider is a major native nocturnal predator, abundant in the *N. attenuata* field site (at the density of 1.55±0. 05 individuals per square meter), which can reduce the survival of *M. sexta* larvae feeding on nicotine-free plants by >40% [1]. We observed that *C. parallela* also preys upon *S. exigua* larvae, in the native habitat; therefore, we examined if *S. exigua* also uses nicotine to deter *C. parallela* and if the observed differences in these two herbivores' susceptibility to *C. parallela* predation could be attributed to their differential nicotine tolerance. Finally, we investigated *C. parallela*'s nicotine metabolism for insights into the nicotine sensitivity of this predator. In short, this work examines the consequences of herbivores' oxidative detoxification of nicotine for its defensive co-option.

## Results

### *Manduca* larvae collected from the field also do not oxidize nicotine

The previously reported experiments on *Manduca*'s nicotine metabolism were conducted with laboratory reared *M. sexta* cultures that were likely to have been inbred and maintained on nicotine-free artificial diet. Given that many insects are known to vary in their detoxification capacities, insects maintained in laboratory cultures on nicotine-free diets may possess different metabolic capabilities than those that feed on nicotine-replete host plants in nature [34], [35]. To test this, we collected eggs of *M. sexta* and its close relative, *M. quinquemaculata* from host plants in Utah and reared the hatched larvae on glasshouse-grown *N. attenuata* plants. Previously, we had developed methods for the efficient extraction (>90%) and quantification of nicotine, cotinine, CNO and NNO by U(H)PLC/ESI-QTOF-MS with detection limits of 0.25 ng nicotine and 0.5 ng cotinine, CNO and NNO [1]. Using these procedures, we analyzed hemolymph and frass of *M. sexta* and *M. quinquemaculata* larvae. None of the reported nicotine oxides could be detected; nicotine was detected in hemolymph [(µg/mL; mean± SE): *M. sexta*- 18.9±1.29, (n = 8); *M. quinquemaculata*- 11.93±4.5 (n = 6)] and frass [Frass (ng/mg; mean± SE): *M. sexta*- 427.35±37.36 (n = 8) *M. quinquemaculata*- 358.85±45.67; (n = 6)].

### Fate and kinetics of nicotine and its oxides in *M. sexta* larvae

**Figure 1:**
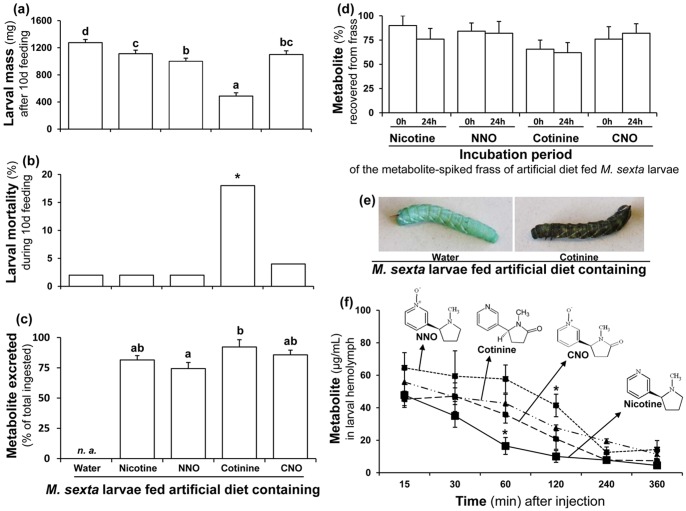
Nicotine oxidation does not benefit *M. sexta* larvae. Larval (**a**) mass [(mean± SE) F_4, 172_ = 45.2, P≤0.05, n = 36, 34, 37, 33 and 37 for water, nicotine, NNO, cotinine and CNO, respectively] and (**b**) mortality (%) after 10 d of feeding on artificial diet containing water (control) or 0.1% (fresh mass) nicotine, NNO, cotinine or CNO (n = 30). (**c**) Waldbauer assay-based quantification of excreted (%) metabolites by fourth-instar larvae fed artificial diet containing 0.1% (fresh mass) metabolite [(mean± SE) F_3, 25_ = 4.3, P≤0.05, n = 6 for nicotine, NNO and cotinine and 8 for CNO]; (*n. a*.≡ not applicable). (**d**) Nicotine, NNO, cotinine or CNO are not degraded in frass over the 24 h period of the Waldbauer assays. Fresh frass was spiked with each metabolite to attain the final concentration of 0.5%; the spiked frass was extracted and analyzed after zero and 24 h of incubation to quantify the recovered metabolite. Every bar represents data from 3 replicates (n = 3). (**e**) Melanization of cotinine-fed (right) larva. (**f**) Discharge kinetics of hemolymph-injected (70±2.5 µg≡ 0.001% of larval fresh mass) nicotine, NNO, cotinine or CNO (n = 5). Lower-case letters and asterisks in (a), (c) and (e) indicate significant differences (*P*≤0.05) by one-way ANOVA; in (b), asterisk indicates significant differences (*P*≤0.05) in frequencies (and displayed percentages) by Fisher's exact test.

To determine whether *M. sexta* can further oxidize ingested nicotine oxides, we fed each nicotine oxide separately to *M. sexta* larvae in an artificial diet. We selected feeding because it is the easiest and the most natural administration method and by feeding one can readily assess the effect of these compounds on larval mass and mortality, from which we could infer if a given oxide was indeed less toxic than nicotine. Mass of *M. sexta* larvae fed artificial diet containing nicotine oxides was not greater than those fed nicotine-diets and larvae fed artificial diet containing NNO and cotinine gained significantly less mass than did nicotine-fed larvae (Fig. 1a). Interestingly, during the 10 d feeding, 18% of the cotinine feeding larvae died (Fig. 1b) and ∼10% melanized (Fig. 1e), suggesting that cotinine is more toxic to *M. sexta* than nicotine. No further oxidized products of the nicotine oxides were found in the larval hemolymph and frass, which contained only the metabolite that the larvae ingested.

We used Waldbauer assays [36] to quantify the flux of ingested nicotine oxides through the larvae, to determine whether nicotine or its oxidation products are metabolized or excreted with different efficiencies. Excretion (%) of the nicotine oxides during the Waldbauer assay was similar to that of nicotine for all compounds tested (Fig. 1c) and these compounds were stable in the frass during the 24 h of assay (Fig. 1d). However, the Waldbauer assay can only evaluate the efficiency of excretion over a 24 h feeding trial, and hence is limited in its abilities to detect differences in the rate of excretion. Hence, to compare the clearance rates of nicotine and nicotine oxides from larval hemolymph, we injected these compounds directly to the hemolymph and periodically measured their concentration in hemolymph until 6 h, when all the metabolites attained their steady clearance rate (Fig. 1f).

### CYP6B46 transcripts are induced by ingestion of nicotine and nicotine oxides but CYP4M1 and CYP4M3 transcripts are not

**Figure 2:**
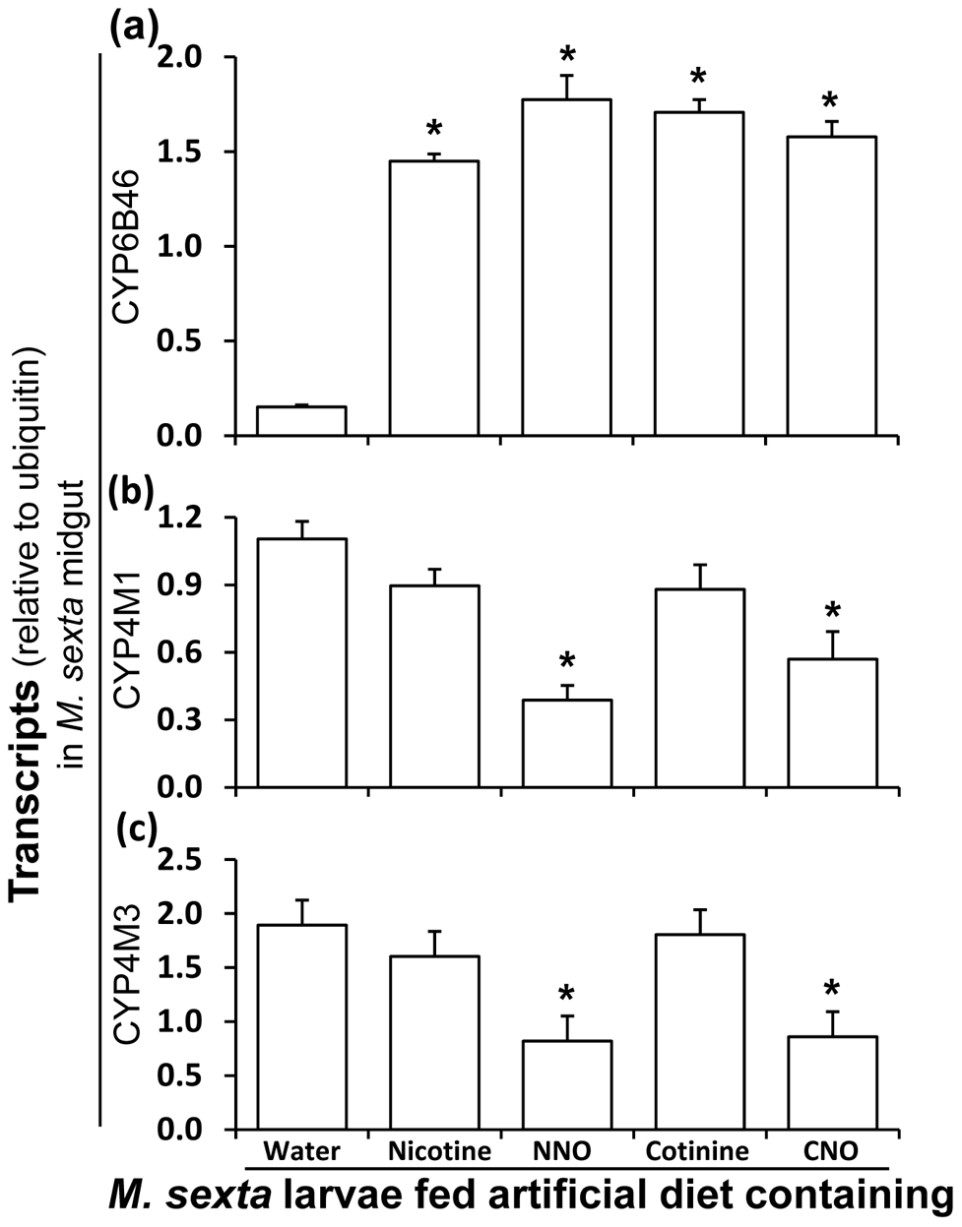
Transcript regulation of *M. sexta* CYPs in response to ingestion of nicotine and nicotine oxides. (**a**) CYP6B46 [(mean± SE) F_4, 19_ = 94.5, P≤0.05, n = 5] (**b**) CYP4M1 [(mean± SE) F_4, 20_ = 9.5, P≤0.05, n = 5] and (**c**) CYP4M3 [(mean± SE) F_4, 20_ = 11.1, P≤0.05, n = 5] transcript levels (relative to ubiquitin) in midguts of 48 h old first-instar *M. sexta* larvae fed artificial diet containing water (control) or 0.1% (fresh mass) nicotine, NNO, cotinine or CNO. Asterisks indicate significant differences (*P*≤0.05) by one-way ANOVA.

We also quantified relative transcript levels of the previously reported nicotine-induced CYP genes in nicotine- and nicotine oxide-fed *M. sexta* larvae. We found that *Ms*CYP6B46 was induced by all the nicotine oxides tested (Fig. 2a), whereas *Ms*CYP4M1 and *Ms*CYP4M3 were not induced by nicotine or its oxides (Fig. 2b and c), results which contrast with previous reports [23], [26].

### *S. exigua* larvae oxidize nicotine

**Figure 3:**
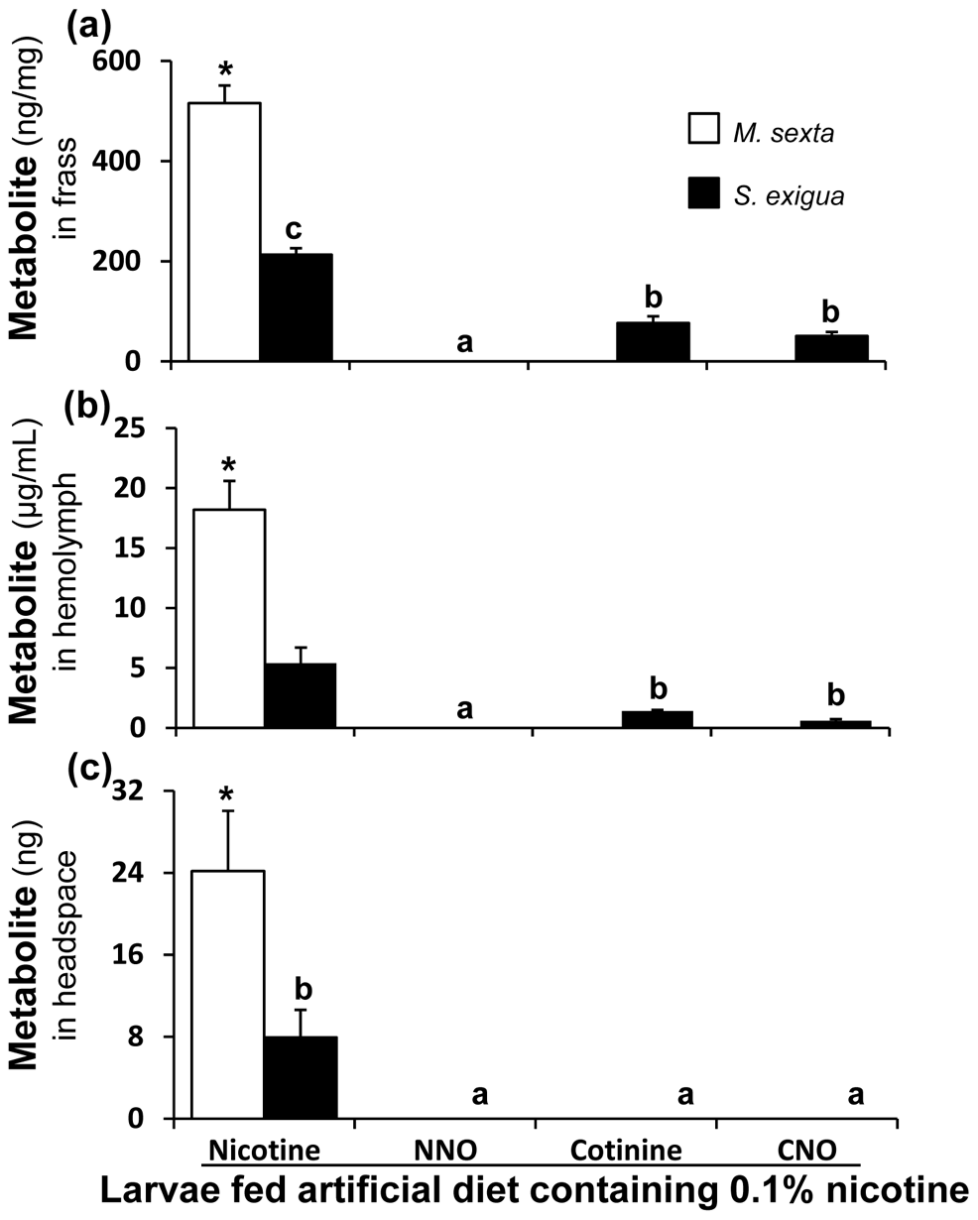
*S. exigua* oxidizes nicotine, but *M. sexta* does not. U(H)PLC/ESI-QTOF-MS-based quantitative analysis of nicotine, NNO, cotinine and CNO in (**a**) frass (**b**) hemolymph and (**c**) headspace of third-instar *M. sexta* (n = 5) and *S. exigua* (n = 5) larvae fed artificial diet containing 0.1% (fresh mass) nicotine. Lower-case letters above the *S. exigua* bars indicate significant differences (*P*≤0.05) among them, by one-way ANOVA. Asterisks above the *M. sexta* nicotine bars indicate that they differ significantly (*P*≤0.05) from the *S. exigua* nicotine values, as determined by one-way ANOVA. Nicotine oxides were not detected in *M. sexta*. The detection limit of nicotine was 0.25 ng and 0.5 ng for cotinine, CNO and NNO; efficiency of extraction was >90% for all these compounds.

**Figure 4:**
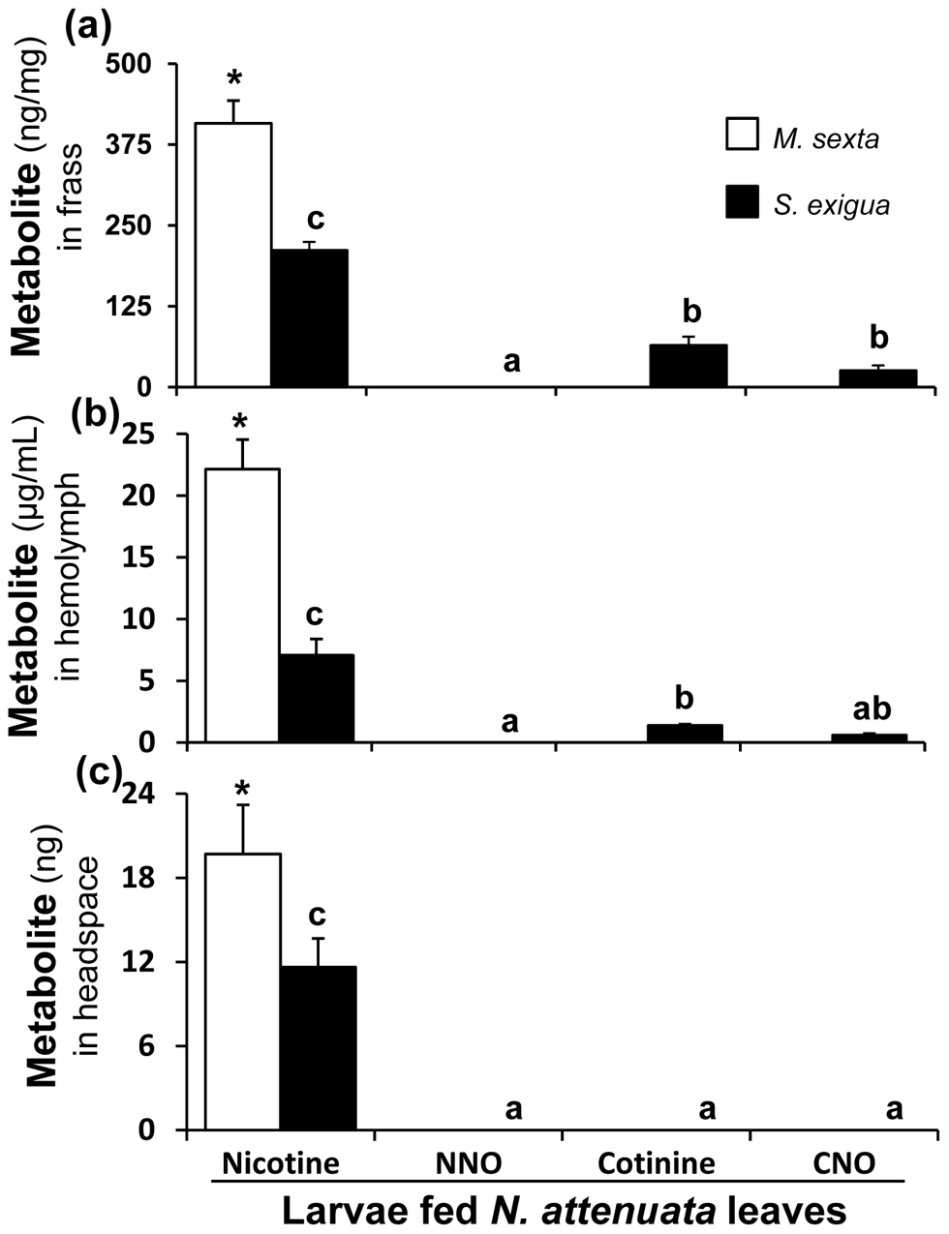
*S. exigua* oxidizes nicotine, but *M. sexta* does not. U(H)PLC/ESI-QTOF-MS-based quantitative analysis of nicotine, NNO, cotinine and CNO in (**a**) frass (**b**) hemolymph and (**c**) headspace of third-instar *M. sexta* (n = 5) and *S. exigua* (n = 5) larvae fed *N. attenuata* leaves. Lower-case letters above the *S. exigua* bars indicate significant differences (*P*≤0.05) among them by one-way ANOVA. Asterisks above the *M. sexta* nicotine bars indicate that they are significantly different (*P*≤0.05) from the *S. exigua* nicotine bars, as determined by one-way ANOVA. Nicotine oxides were not detected in *M. sexta*. The detection limit of nicotine was 0.25 ng and 0.5 ng for cotinine, CNO and NNO; efficiency of extraction was >90% for all these compounds [1].

Quantitative analysis of nicotine metabolites in the frass, hemolymph and headspace of artificial diet (Fig. 3a–c) and *N. attenuata*-fed (Fig. 4a–c) *S. exigua* larvae was conducted to understand the nicotine metabolism in this species. In addition to nicotine, all three nicotine oxides reported by Snyder *et al*. [24] (NNO, cotinine and CNO) were found in the frass; cotinine and CNO were less than 50% as abundant as nicotine, whereas NNO was present only in trace amounts (detection limits: nicotine- 0.25 ng; cotinine, CNO and NNO- 0.5 ng) (Fig. 3a; Fig. 4a). In the hemolymph, cotinine and CNO were also less than 50% as abundant as nicotine but NNO was not detected (Fig. 3b; Fig. 4b). The amounts of nicotine found in *S. exigua* larval frass, hemolymph and headspace were significantly lower than those found in the respective samples of *M. sexta* larvae (Fig. 3a–c; Fig. 4a–c).

**Figure 5:**
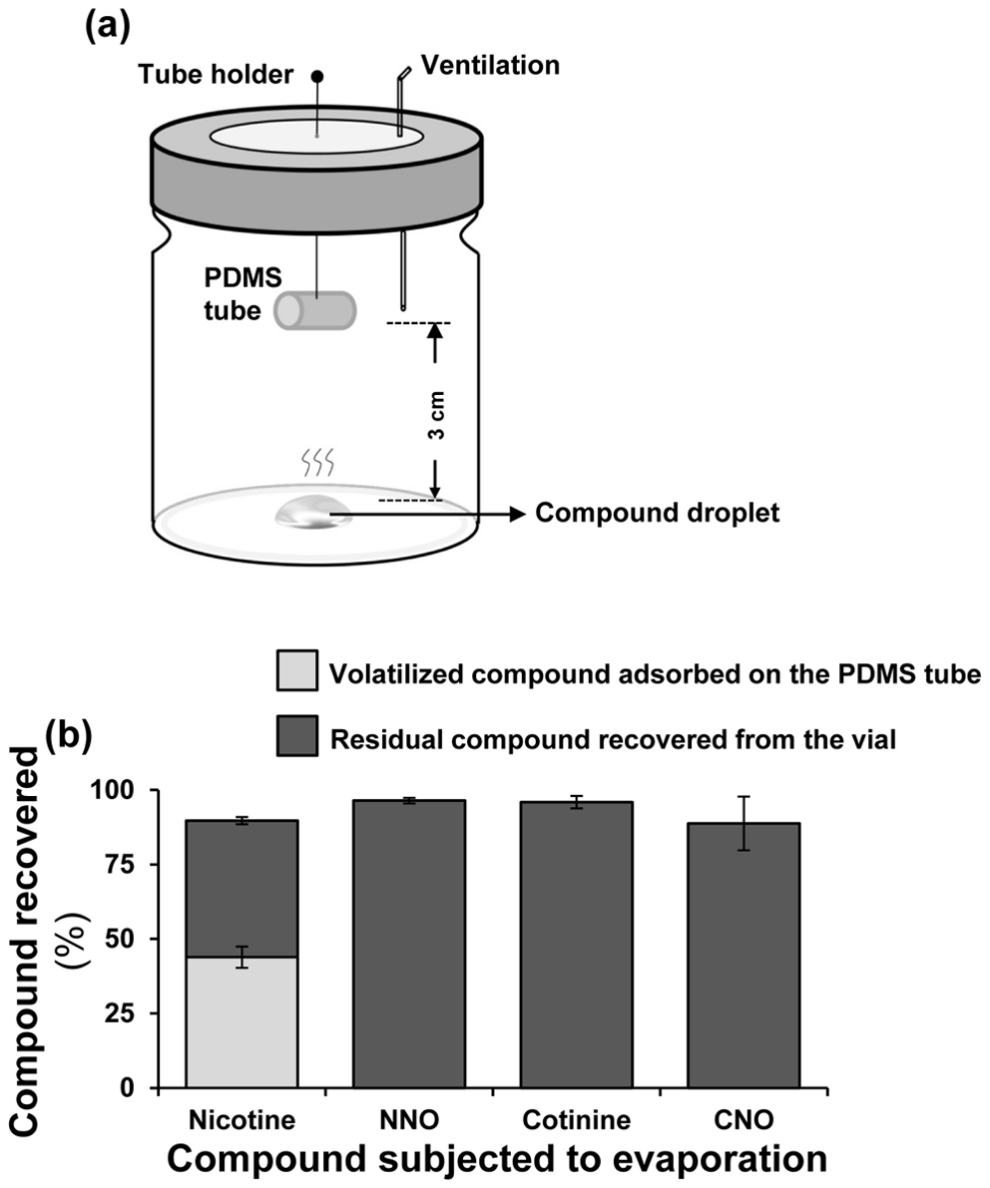
Volatility analysis of nicotine and nicotine oxides. (**a**) Schematic of setup used for the collection of evaporated nicotine, NNO, cotinine and CNO placed in the collection vial (1 µg/5 µL methanol). (**b**) Percentage of evaporated and residual nicotine, NNO, cotinine and CNO (n = 4); one µg of nicotine, NNO, cotinine or CNO (in 5 µL methanol) was incubated for 1 h.

Nicotine was the only metabolite found in the headspaces of all larvae (Fig. 3c; Fig. 4c). We analyzed and compared the volatility of nicotine oxides to that of nicotine as described by Kumar *et al*. [1] (Fig. 5a–b). Most of the cotinine, CNO and NNO used in these volatility assays was recovered from the walls of the assay-vials and not detected in the polydimethylsiloxane (PDMS)-adsorbed headspace samples.

### Nicotine is more toxic than the nicotine oxides to *S. exigua*

**Figure 6:**
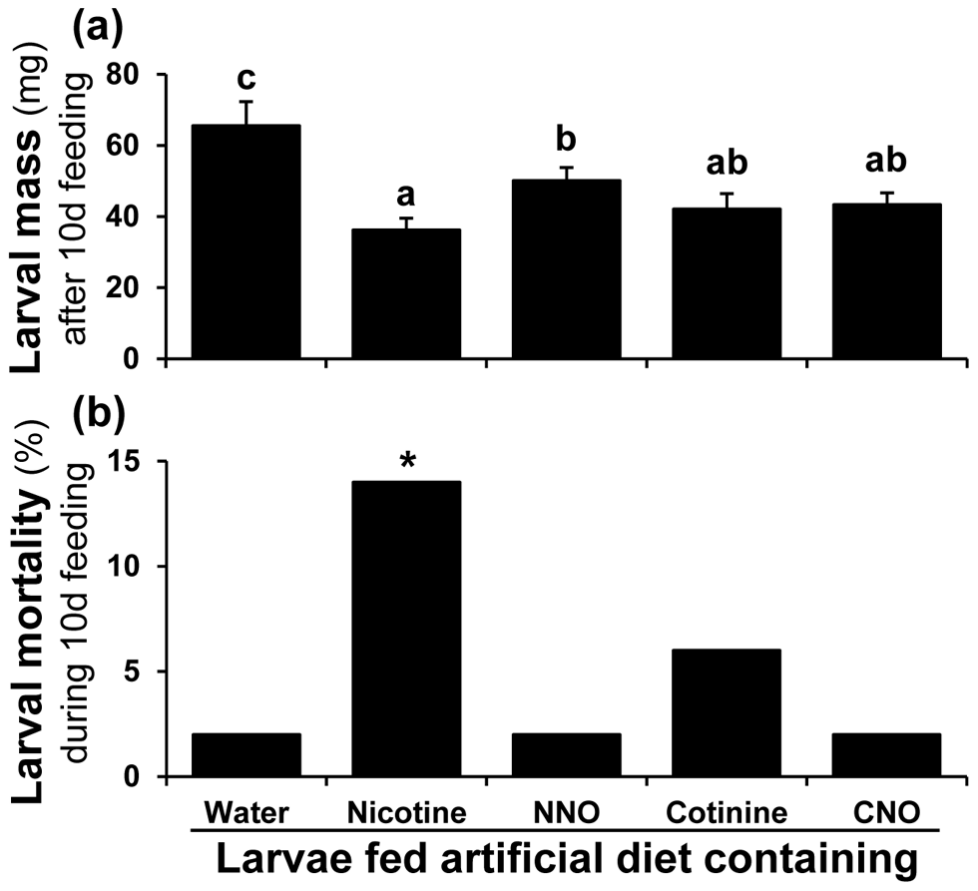
Nicotine is more toxic than the nicotine oxides to *S. exigua* larvae. (**a**) Larval mass of *S. exigua* [(mean± SE) F_4, 154_ = 6.03, P≤0.05, n = 32, 27, 33, 30 and 33 for water, nicotine, NNO, cotinine and CNO, respectively] and (**b**) mortality (%) of *S. exigua* larvae during 10days of feeding artificial diet containing water (control) or 0.1% (fresh mass) nicotine, NNO, cotinine or CNO; each bar represents data from 30 larvae. In (a), lower-case letters above the bars indicate significant differences (*P*≤0.05) by one-way ANOVA; in (b) asterisk indicates significant differences (*P*≤0.05) in frequencies (and displayed percentages) by Fisher's exact test.

*S. exigua* larvae fed NNO-containing diet grew significantly larger than those fed cotinine-, CNO-, or nicotine-containing diets (Fig. 6a). Nicotine feeding caused the highest mortality (14%) of all metabolites tested in *S. exigua* larvae (Fig. 6b).

### Nicotine oxidation in *S. exigua*

Cotinine, CNO, and NNO were found in the hemolymph and frass of nicotine-fed *S. exigua* larvae. CNO was also detected in the hemolymph and frass of cotinine-fed larvae. However, CNO was not found in the NNO-fed larvae and NNO was not found in the cotinine-fed larvae, suggesting that in *S. exigua*, cotinine and NNO are formed from nicotine and CNO is formed from cotinine.

### Nicotine but not its oxides deter *C. parallela*

**Figure 7:**
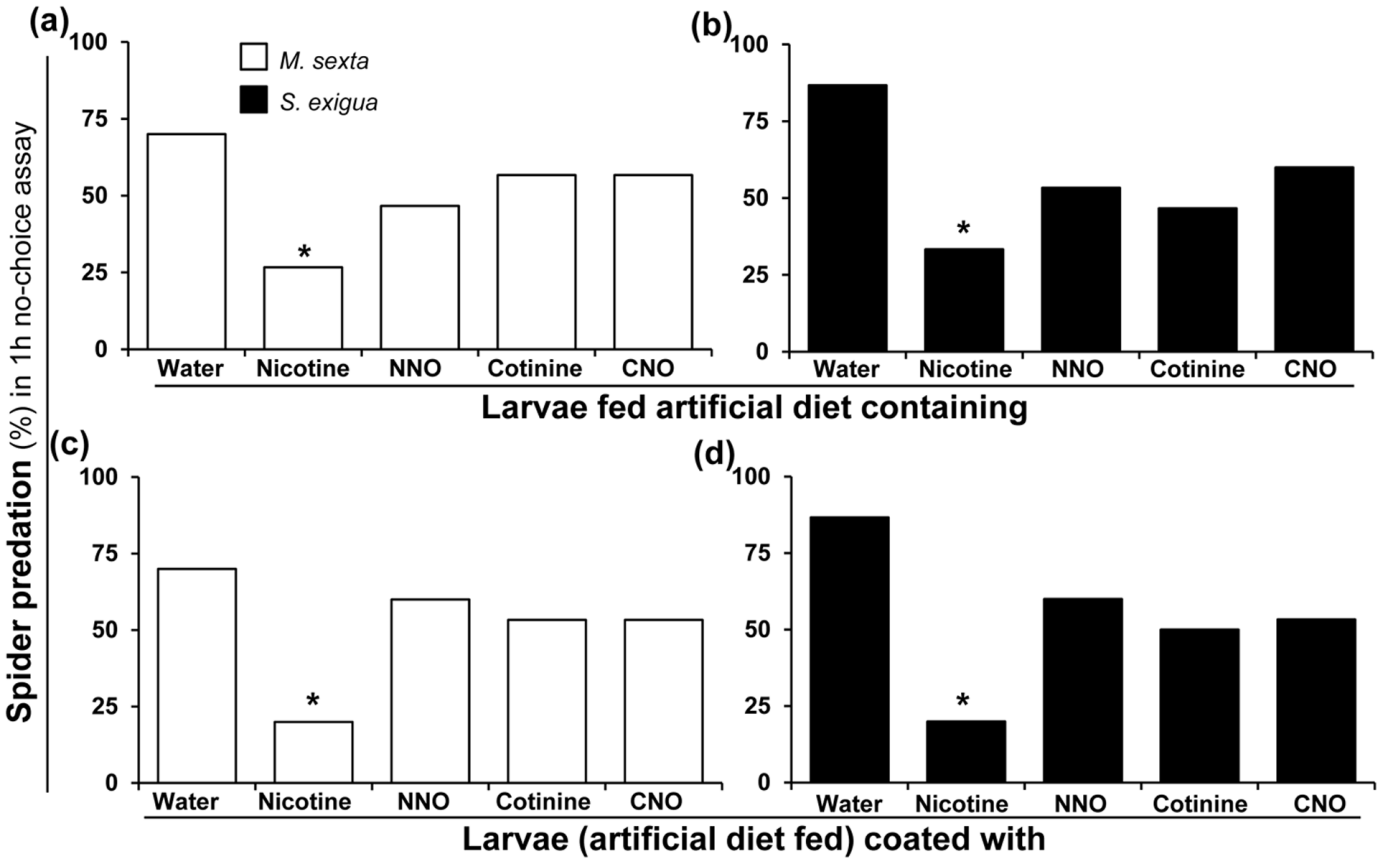
Nicotine deters *C. parallela* but nicotine oxides do not. *C. parallela*'s predation (%) (in 1 h no-choice assay) on second-instar (**a**) *M. sexta* and (**b**) *S. exigua* larvae fed artificial diet containing water (control) or 0.1% (fresh mass) nicotine, NNO, cotinine or CNO. *C. parallela* predation (%) (in 1 h no-choice assay) on second-instar artificial diet fed (**c**) *M. sexta* and (**d**) *S. exigua* larvae coated with water (control) or 0.2% aqueous nicotine, NNO, cotinine or CNO. Each bar represents data from 30 larvae. Asterisks indicate significant differences (*P*≤0.05) in frequencies (and displayed percentages) by Fisher's exact test.

Previously, we demonstrated that nicotine exhaled by *M. sexta* larvae deters *C. parallela* [1]. Here, we tested whether nicotine oxides also deter *C. parallela* and compared their deterrence- effects to that of nicotine. We conducted no-choice *C. parallela* predation assays using *M. sexta* and *S. exigua* larvae that were fed or coated with nicotine or its oxides. *C. parallela* were not deterred by NNO-, cotinine-, or CNO-fed/coated *M. sexta* or *S. exigua* larvae (Fig. 7a–d). Nicotine-coated *S. exigua* larvae were only half as likely to be eaten as nicotine-fed ones. In contrast, this difference was not observed between nicotine-coated and nicotine-fed *M. sexta* larvae.

**Figure 8:**
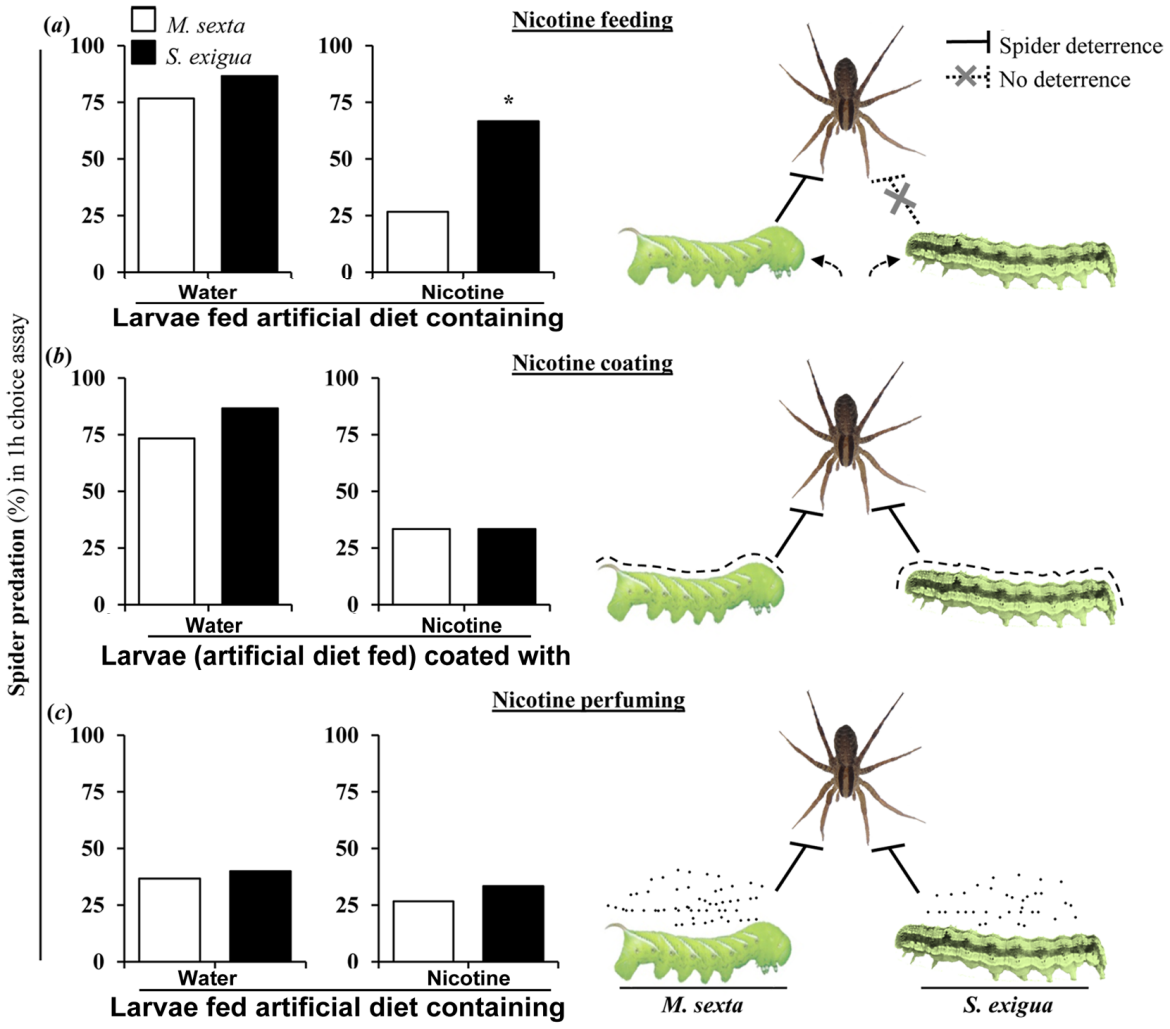
*C. parallela*'s preference of *S. exigua* larvae over *M. sexta* larvae is diminished by topically coating larvae or supplementing their headspace with nicotine. *C. parallela*'s predation (%) (in 1 h choice assay) on second-instar *M. sexta* and *S. exigua* larvae fed (a) artificial diet or artificial diet containing 0.1% (fresh mass) nicotine (b) artificial diet and coated with water (control) or nicotine, and (c) artificial diet containing water (control) or 0.1% (fresh mass) nicotine and having the assay environment nicotine-perfumed using 500 µL of 1 mM nicotine on a cotton swab. Schematics in right panels of (a), (b) and (c) show the effects of various modes of nicotine supplementation to *M. sexta* and *S. exigua* larvae on the spider predation behavior. Each bar represents data from 30 larvae. Asterisks indicate significant differences (*P*≤0.05) in frequencies (and displayed percentages) by Fisher's exact test.

In choice assays, we found that *C. parallela* preferred nicotine-fed *S. exigua* larvae over nicotine-fed *M. sexta* larvae (Fig. 8a). This preference diminished when nicotine was topically applied to the larvae (Fig. 8b) or when nicotine was perfumed in the assay containers (Fig. 8c). Cotinine, CNO and NNO perfuming did not affect *C. parallela* predation.

### *C. parallela* does not oxidize nicotine

Since nicotine strongly deterred *C. parallela*, we evaluated its nicotine metabolism by analyzing its frass. *C. parallela*'s frass contained only nicotine [68.9±18.9 (ng/mg; mean± SE) (n = 5)], suggesting that *C. parallela* is unable to oxidize and detoxify nicotine by the known means of oxidizing nicotine.

## Discussion

Recently, in a race of the polyphagous aphid, *Myzus persicae* that thrives on cultivated tobacco, Bass *et al*. [28] conclusively demonstrated that nicotine is oxidatively detoxified. Such conclusive evidence of oxidative detoxification *M. sexta* has been lacking despite more than 50 years of research. We conducted an unbiased examination of *M. sexta*'s nicotine metabolism and obtained results consistent with the rapid excretion theory but not with the oxidative detoxification theory. Oxidative nicotine metabolites were not found in larvae from *M. sexta* laboratory colonies, collections from nature, and the closely related species, *M. quinquemaculata*. Differences from the results presented here and previous studies reporting nicotine oxidation in *M. sexta* could be ascribed to differences in experimental design and methods. Snyder *et al*. [24] attributed their success in detecting CNO to the highly advanced HPLC technology of their time and predicted that the use of technologies such as LC-MS-MS would provide further clarity. We did not detect evidence of any known nicotine oxides with U(H)PLC/ESI-QTOF-MS technology. We used third-instar larvae in contrast to the fifth-instar larvae used in the previous studies, which might differ physiologically from the first four growth-focused instars, as during the fifth instar, larvae prepare for pupation [21], [24]. Similar to the work of Self *et al*. [29], we always fed the larvae immediately after eclosion on physiologically realistic concentrations of dietary nicotine (up to 0.1% of diet fresh mass, levels found in *N. attenuata* leaves), whereas Snyder *et al*. [24] and Wink and Theile [21] used the artificial diet-reared fifth-instar larvae, which had not been exposed to nicotine during their previous instars; these larvae were then abruptly exposed to high and physiologically unrealistic amounts of nicotine (0.75 and 1.0% of fresh diet, respectively). We used freshly collected frass and hemolymph, whereas previous investigators used stored and dried frass in which nicotine oxidation may have occurred spontaneously [24]. Lastly, we ruled out the possibility that nicotine was oxidized by the chemical constituents of diet, before it was ingested by the larvae, a possibility not examined in the previous work.

None of the nicotine oxides were discharged from the hemolymph more rapidly than nicotine, clearly rejecting the long standing hypothesis that being more polar than nicotine, the oxides are more rapidly excreted [24], [37]. *M. sexta* larvae use pumps to purge toxic alkaloids to protect their nervous systems; these pumps function with structurally similar alkaloids like nicotine, morphine, atropine or even alkaline synthetic dyes [30], [38]. Previously, we proposed that *Ms*CYP6B46 could be part of such a pump [1]. The Waldbauer assays results were consistent with a ‘purge by excretion’ mechanism that functions with comparable efficiency between nicotine and its oxides. *Ms*CYP6B46 transcripts were induced in larval midguts by the ingestion of all nicotine oxides, but no further oxidized products of nicotine, NNO or cotinine were found in the hemolymph or frass. Collectively, these results demonstrate either the absence of CYP-mediated nicotine oxidation mechanisms in *M. sexta*, or that *Manduca*'*s* metabolism of nicotine is so rapid and efficient that no oxidative intermediates accumulate, or involves a novel pathway.

All of the nicotine oxides had similar effects on larval growth as nicotine did and none deterred *C. parallela*, indicating that the oxidation of nicotine is not advantageous for *M. sexta* and hence cannot be considered either a detoxification or a co-option related process. The nicotine-elicited *Ms*CYP6B46 clearly provides an ecological benefit for the larvae, due in large part to the volatility of nicotine [1]. *M. sexta* larvae would clearly be at an ecological disadvantage if they converted nicotine to a less volatile and consequently, less spider-deterrent oxides. In contrast, for *S. exigua*, which is more sensitive to *N. attenuata*'s nicotine than *M. sexta* [19], oxidizing nicotine to cotinine, which is less lethal, represents true detoxification. However, since cotinine and the other two nicotine oxides are not emitted in the headspace, probably due to their lower volatilities, this mode of detoxifying nicotine represents a loss of defensive utility, at least against *C. parallela* predation (Fig. 3c and Fig. 4c). Nicotine-coated *S. exigua* larvae deterred *C. parallela* more than those which ingested nicotine, suggesting that the externalized nicotine is more effective than the externalized nicotine oxides. *M. sexta* does not oxidize nicotine and repurposes three times more nicotine than does *S. exigua* into its hemolymph which can be externalized to repel predators. In *N. attenuata*'s niche, nicotine is an important xenobiotic, which even affects the members of the third and fourth trophic levels [1], [39], [40]. Nicotine detoxification by *S. exigua* reduces its transfer to the next trophic levels, which likely explains why *C. parallela* prefers these larvae over *M. sexta* as prey.

*Lycosa ceratiola* and *Nephila clavipes* spiders are deterred by pyrrolizidine alkaloids sequestered by the aposematic *Utetheisa ornatrix* moths [41], [42]. However the basis of their sensitivity to the alkaloids remains unknown as does their ability to metabolize the alkaloids. *C. parallela* do not appear to oxidize nicotine; whether this inability renders them nicotine-sensitive and whether the lower toxicity of nicotine oxides makes *C. parallela* less sensitive, remain open questions. Nicotine exhalation process is an atypical example of olfactory aposematism, in that: 1) the toxin is not sequestered (only 0.6% of ingested nicotine is rerouted into the hemolymph by the action of CYPB646) and is released only while the herbivore is ingesting it; and 2) the aposematic signal is conspicuous only to a specific predator [1], [43]. Our results show that nicotine oxidation reduces the volatility of defensive signal, which limits the deterrence-potential of this spiracular emanation. We infer that by oxidizing nicotine, *S. exigua* renders itself less-aposematic to at least, the ground-dwelling *C. parallela*; *S. exigua* larvae which are known to drop from their host plants after sensing the vibrations of flying insects with their sensory hairs [44], are likely easy prey for these spiders.

The susceptibility of insect herbivores to natural enemies varies as a function of larval host plant and plant-mediated protection is thought to favor host plant specialization [45], [46]. Bernays and Graham [45] hypothesized that generalist predators primarily consume generalist prey and become important factors in selecting for narrower diet breadth in polyphagous insects; *C. parallela*'s preference of *S. exigua* is consistent with this hypothesis. *M. sexta* and *S. exigua* are generally considered to be (Solanaceae-) specialists and generalists, respectively; however, they would be inappropriate models for the analysis of the evolution of ‘generalist’ and ‘specialist’ nicotine-metabolism-strategies, given that they are phylogenetically distant [47]. However in the context of *M. sexta*'s exceptional nicotine tolerance and its co-option of this plant defense by exhalation and the nicotine oxidation by *S. exigua*, this pair of species have traits consistent with the suppressed xenobiotic metabolism and co-option by the specialists and the canonical ‘spontaneous oxidative response’ of generalists that theory predicts [6], [7], [48]. It would be interesting to study, whether nicotine has been a bottom-up and *C. parallela* a top-down selection force in *M. sexta*'s host-specialization. On the other hand, as seen in some generalist herbivores [12], [13], [14], [15], constitutive or induced pharmacophagy to acquire nicotine was not observed in *S. exigua*. This is presumably because *S. exigua* has only recently been introduced in USA (first reported in 1876 in Oregon) [49].

In summary, oxidative nicotine detoxification is not responsible for the exceptional nicotine tolerance of *M. sexta*, which gains an ecological advantage against predatory spiders by not metabolizing nicotine. Spider deterrence is a function of nicotine's volatility; this is lost by *S. exigua* while detoxifying, as it detoxifies nicotine to non-volatile oxides. Hence, the results are consistent with hypothesis that generalist herbivores incur greater costs of detoxification, with the costs being more ecological in nature, than the much-discussed energetic, physiological and metabolic costs.

## Materials and Methods

### Chemicals

Nicotine and cotinine were purchased from Sigma-Aldrich (Germany). CNO and NNO were synthesized as previously reported [50], [51].

### Artificial diet

Artificial diet for feeding *M. sexta* and *S. exigua* larvae was prepared according to Grosse-Wilde *et al* [52]. Artificial diet containing 0.1% (fresh mass) nicotine, cotinine, NNO or CNO was prepared by adding an aqueous solution (100 mg/mL) of each metabolite to the diet, followed by thorough hand-mixing. Artificial diet mixed with similar amount of water was used as control.

### Plant material

*N. attenuata* plants were grown, as previously reported by Krügel *et al*. [53].

### Insects

*M. sexta* eggs were obtained from an in-house colony [52]. Eggs of *M. sexta* and *M. quinquemaculata* were also collected from *N. attenuata* or *Datura wrightii* plants in the field [the Great Basin desert Utah, USA (37°08′45″N, 114°01′11″W)]. *S. exigua* eggs were procured from Benzon Research (USA). All larvae were reared on artificial diet or *N. attenuata* leaves, in a growth-chamber (Snijders Scientific, The Netherlands), at 26°C/16 h light: 24°C/8 h dark. Neonates were fed their respective diets until they were used in experiments. Every day, the larvae were provided with their respective fresh diets.

### Analysis of nicotine, NNO, cotinine and CNO from AD, larval frass and hemolymph

Nicotine and nicotine oxides from frass and hemolymph of third-instar *M. sexta* and *S. exigua* larvae fed artificial diet, artificial diet containing (0.1%) nicotine, NNO, cotinine or CNO, or *N. attenuata* plants were detected and quantified by U(H)PLC/ESI-QTOF-MS with a Phenomenex Gemini NX 5 column (5×2.0 mm; particle size 3 µM), as reported previously [1]. Extraction buffer containing internal standards (1 ng/µL) nicotine-d_3_, NNO-d_3_, cotinine-d_3_ and CNO-d_3_ was used for all the extractions.

To test if nicotine, NNO, cotinine or CNO were degraded or oxidized in the frass, after they were excreted and exposed to the environment, these metabolites were separately spiked onto fresh frass [0.5% in 50 mg (fresh mass) frass] which was subsequently incubated for 24 h at the above mentioned insect rearing conditions; 0 h incubated samples were used as controls. These frass samples were extracted and the metabolites were quantified, as described previously [1]; no degradation or further oxidation of nicotine or its oxides was observed (Fig. 1d).

Similarly, we spiked nicotine in artificial diet (2 µg/mg; n = 5), incubated for 0 and 24 h and examined if the chemical components of artificial diet oxidized nicotine. No oxides were detected in the extracts of 0 and 24 h incubated samples, confirming the absence of oxidative components in artificial diet; all the spiked nicotine was recovered from artificial diet, a result confirming its stability in artificial diet.

### Analysis of nicotine and nicotine oxides in larval headspace

We examined whether the (*M. sexta* and *S. exigua*) larvae ingesting nicotine exhaled NNO, cotinine and CNO, as *M. sexta* larvae exhales nicotine; volatile trapping, quantification and the determination of trapping and quantification efficiencies were performed as described previously [1]. *M. sexta* as well as *S. exigua* larvae of 100±10 mg (fresh mass) were used in these analyses. Larvae fed artificial diet, artificial diet containing 0.1% nicotine or *N. attenuata* leaves were incubated for 1 h, in a sealed and ventilated glass vial (5cc) to trap their exhalations on a piece of adsorptive PDMS tube [2 mm length (Reichelt Chemietechnik, Germany)] suspended from the seal of the vial. PDMS-adsorbed nicotine or nicotine oxides were extracted using 50 µL extraction buffer. Extracted metabolites were analyzed and quantified by U(H)PLC/ESI-QTOF-MS.

### Volatility analysis of nicotine and nicotine oxides

Volatilities of nicotine oxides were measured by the method previously developed for nicotine's volatility analysis [1]. One µg of nicotine, NNO, cotinine or CNO (in 5 µL methanol) was incubated (n = 4) in a sealed glass vial (5cc); five µL methanol was used as a negative control. Using a solid needle, a PDMS tube was attached to the vial's lid so as to remain suspended in the sealed vial (Fig. 5a). An injection needle (0.08×40 mm, BRAUN, Germany) was inserted through the lid for ventilation. This setup was incubated for 1 h at 30°C. Compounds adsorbed on the PDMS tubes were extracted using 50 µL extraction buffer. Residual nicotine or nicotine oxide in the vial was also extracted after 1 h incubation, using 500 µL extraction buffer. Metabolites extracted from PDMS and vials were quantified by U(H)PLC/ESI-QTOF-MS.

### Waldbauer assays

Ingestion and excretion of nicotine and nicotine oxides by *M. sexta* larvae were budgeted using Waldbauer assays [1], [36], [54]. *M. sexta* neonates were transferred to artificial diet containing 0.1% (fresh mass) of nicotine, cotinine, NNO or CNO (n = 50). Larvae were reared on these diets for 13 d. As described previously [1], on day 14 larvae were weighed and then starved for 4 h to empty their guts and clear previously ingested metabolites from their bodies. Starved larvae were weighed again and fed for 24 h on their respective diets. After feeding, the larvae were again weighed before and after the second 4 h starvation. Remaining diet and the frass excreted by larvae during the 24 h feeding and second starvation were weighed. The amount of metabolite ingested by each larva was calculated as 0.1% of the diet it consumed. The collected frass was analyzed for its metabolite content by U(H)PLC/ESI-QTOF-MS. Percentage of excreted metabolite was determined as the amount of metabolite excreted/amount of metabolite ingested ×100 [36].

### Excretion kinetics of nicotine and nicotine oxides

Discharge rates of nicotine and nicotine oxides from the hemolymph of *M. sexta* larvae were measured after injecting them separately into the larval hemolymph. Injected metabolites in hemolymph were quantified by U(H)PLC/ESI-QTOF-MS from repeated sampling of the hemolymph. Fifty µL water (control) or aqueous nicotine, cotinine, NNO or CNO containing 70±2.5 µg compound was injected into the hemolymph from the dorsal point between 5^th^ and 6^th^ body segments of previously CO_2_-anesthetized fourth-instar larvae (7.0 g±0.25 g fresh mass), to attain 0.001% of fresh mass concentration of each metabolite. Hemolymph (2 µL) of each larva was collected at 0, 30, 60, 180, and 360 min by clipping the tip of the horn [1].

### Performance of larvae feeding nicotine or nicotine oxide containing diet

*M. sexta* and *S. exigua* neonates (n = 50) were reared on artificial diet containing water (control) or 0.1% (fresh mass) nicotine, cotinine, NNO or CNO. On the 10^th^ day, surviving larvae were counted and their masses recorded.

### RNA isolation and real time quantitative PCR

*M. sexta* neonates were fed artificial diet containing water (control) or 0.1% (fresh mass) nicotine, cotinine, NNO or CNO for 24 h. Larvae were dissected and their midguts were stored in Trizol (Invitrogen, Germany), at 4°C until further use. Midgut RNA was isolated following the manufacturer's instructions. cDNA was synthesized over this RNA and a quantitative real time PCR was performed using this cDNA to measure the levels of *Ms*CYP6B46, *Ms*CYP4M1 and *Ms*CYP4M3 transcripts, as reported previously [55]. Ubiquitin transcripts were used as an internal control to normalize the abundance of *Ms*CYP6B46, *Ms*CYP4M1 and *Ms*CYP4M3 transcripts.

### Coating *M. sexta* and *S. exigua* larvae with nicotine or its oxides

Artificial diet fed *M. sexta* or *S. exigua* larvae (50±5 mg fresh mass) were coated with 5 µL water (control) or 0.2% aqueous nicotine, NNO, cotinine or CNO, using a 10 µL micropipette tip.

### Spider predation (choice and no-choice) assays

*C. parallela* spiders were collected from their native habitat (the Great Basin desert Utah, USA). They were starved for 12 h before the assays. Spider predation (choice, no-choice and no-choice with perfuming) assays were performed as previously described [1]. Second-instar *M. sexta* and *S. exigua* larvae, fed artificial diet containing nicotine or its oxides or coated with these compounds were used in these assays. In choice assays, each *C. parallela* individual was provided with one test and one control larva, in a polypropylene container (60cc) and was allowed only one choice during the 1 h assay period. *C. parallela*'s choices were expressed in terms of percentage of *C. parallela* that chose larvae from each treatment group. In no-choice assays, each *C. parallela* was provided only one larva (test or control). Assays with test and respective control larvae were always performed simultaneously. Percentage of larvae from each treatment group that *C. parallela* preyed upon in 1 h was calculated and used as a measure of *C. parallela*'s predation preference. No-choice assays with perfuming were performed similar to the no-choice assays, except that a perforated parafilm covered Eppendorf tube containing a cotton swab moistened with nicotine, cotinine, CNO or NNO (500 µL; 1 mM) or water (500 µL; control) was placed in each container, during the assays, as described by Kumar *et al*. [1].

### Analysis of *C. parallela*'s excreta

*C. parallela* were allowed to prey on second-instar *M. sexta* larvae that were fed artificial diet or artificial diet containing 0.1% nicotine (fresh mass). *C. parallela*'s excreta (3 mg) were extracted in 100 µL extraction buffer. Extracts were analyzed by U(H)PLC/ESI-QTOF-MS [1].

### Statistical analyses

All the quantitative data (metabolites, hemolymph, frass and headspace, CYP transcript levels and larval mass) were subjected to one-way ANOVAs and the statistical significance (P≤0.05) was determined by Fisher's least significant difference *post hoc* test. Significance (*P*≤0.05) of the binary results (mortality of larvae feeding artificial diet containing various metabolites and spider predation) was evaluated with Fisher's exact test. For each column in the figures, the data were normalized to their percentages to facilitate visual comparisons. Percentages were also analyzed by the Fisher's exact test and the significance applicable to both frequencies and percentages are shown in the figures.
